# Egocentrically-stable discriminative stimulus-based spatial navigation in mice: implementation and comparison with allocentric cues

**DOI:** 10.1038/s41598-019-42852-0

**Published:** 2019-04-23

**Authors:** Jinsung Chun, Youngsoo Kim, Jin Woo Choi, Daesoo Kim, Sungho Jo

**Affiliations:** 1 School of Computing, Korea Advanced Institute of Science and Technology (KAIST), 291, Daehak-ro, Yuseong-gu, Daejeon, 34141 South Korea; 2 Graduate School of Medical Science and Engineering, KAIST, Daejeon, 34141 South Korea; 3 Department of Biological Sciences, KAIST, Daejeon, 34141 South Korea

**Keywords:** Spatial memory, Animal behaviour

## Abstract

Animals are capable of using visual cues to find the correct route during navigation. These visual cues, which contain spatial information on the direction towards the goal point, are perceived either allocentrically or egocentrically. In this study, we examined how navigating with these two types of visual cues affects the learning processes of rodents. To present egocentrically-stable spatial cues, we devised a head-mounted device that provided discriminative orientation cues that indicated the correct choice at a fork within a double Y-maze. For allocentrically-stable spatial cues, LEDs serving as external route-mark cues were attached to the walls of the double Y-maze and illuminated to indicate the correct pathway. To rule out the possibility of the mice using extra-maze cues, we rotated the entire maze and used different start and goal sites for every trial. Our results revealed that mice using egocentric cues and external route-mark cues both showed a sigmoidal learning process for spatial navigation and that external route mark-based learning, surprisingly, learned faster than egocentric stimulus-based learning in egocentric space.

## Introduction

Animals rely on information from their surroundings to correctly navigate to a destination. Rodents, for example, utilize spatial cues from their environment for two navigational strategies: a locale strategy and a taxon strategy^1^. Locale strategies are based on creating cognitive maps with spatial information from the environment to guarantee a stable and flexible navigation strategy after the construction of map-based spatial memory. Taxon strategies, on the other hand, code route instructions into a list of stimulus-response commands and ensures successful navigation of the subject as long as the given instructions on the route towards the goal location are reliable and sufficient.

Despite both types of navigational strategies requiring spatial cues, the information provided by the cues for the two strategies manifests different properties. Locale strategies use allocentric cues, or cues that are independent from the orientation of the subject. Landmarks or road signs are examples of allocentric cues; the information they provide does not depend on the direction the subject is facing. Taxon strategies use cues that provide information dependent on the orientation of the subject. Head-up displays used for automobiles and beacons exemplify such cues.

Previous studies have provided evidence for animals using visually given cues to navigate, such as honey bees navigating with visual cues^2,3^. However, there has not been an acceptable mammalian model for studying egocentric navigational behavior resulting from a taxon strategy. Although there has been meaningful research on controlling the movement of rodents in egocentrically stable space using artificial somatosensory signals associated with the stimulation of a reward pathway^4^, using artificial somatosensory signals is inadequate for comparing the performance of learning with allocentric cues and the performance of learning with egocentric cues, as somatosensory signals are inherently egocentric. In this study, we devised an experimental paradigm reflecting the innate properties of learning with either allocentrically stable or egocentrically stable stimuli in mice. Food was supplied at goal locations as a delayed reward after an appropriate sequence of foraging behavior.

In this research, we analyzed the learning procedures of the two navigational paradigms by utilizing visually presented cues that are stable in different spaces. To compare the learning using the two aforementioned navigational strategies, a specialized experimental paradigm in which experimenters determine whether animals perceive the two different types of visual cues as route instructions in taxon system was taken in consideration. Moreover, the types of spatial instructions were classified as the allocentrically-stable cues (external route-marks indicating the correct route towards the goal location) and egocentrically-stable cues (egocentric discriminative stimuli indicating correct directions). Inspired by the mechanism of navigation devices and based on the basic abilities of rodents in maze learning^5,6^, we devised a novel experimental paradigm, in which mice find a short-cut to the goal using external route-marks and egocentric stimuli as allocentrically and egocentrically given cues for taking a left or right in a specialized maze.

## Results

### Double Y-maze paradigm for egocentric stimulus-based learning

**Figure 1:**
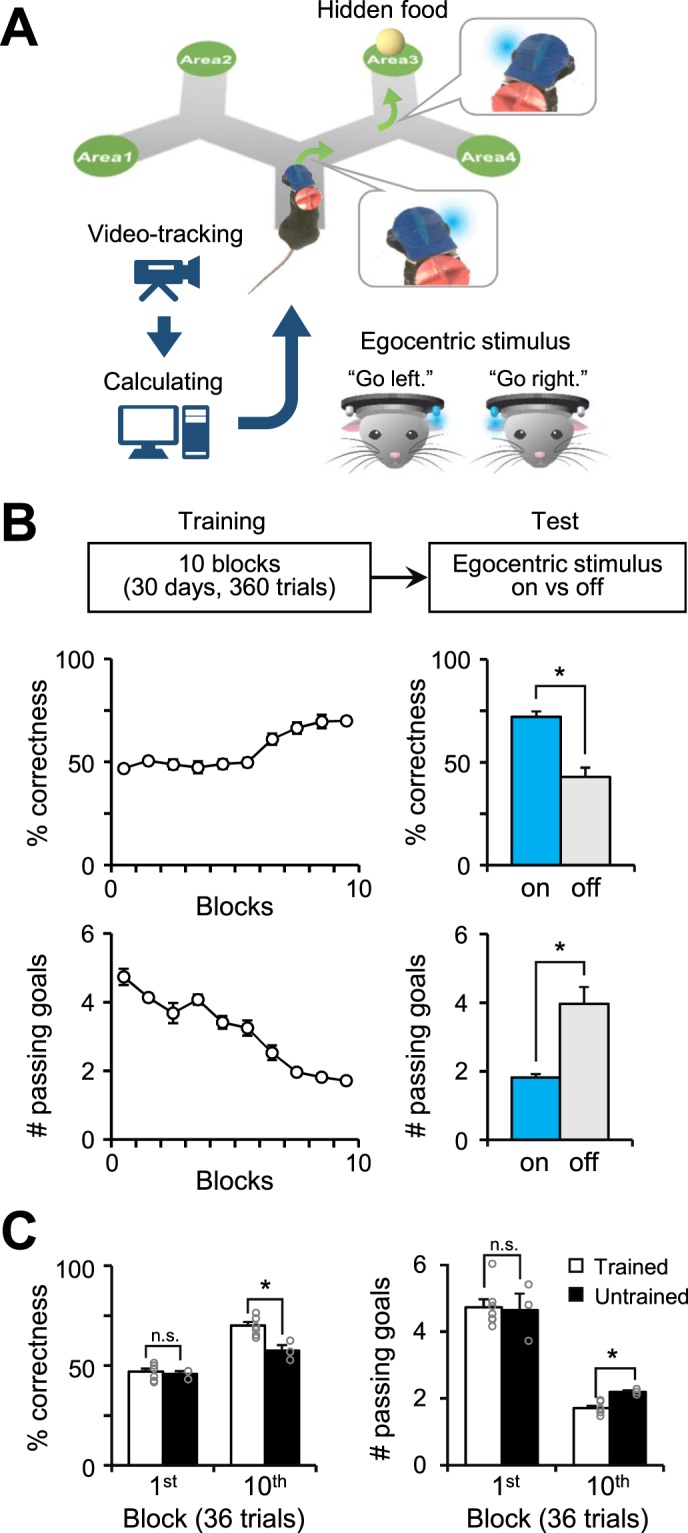
Mice successfully showed egocentric stimulus-based learning for spatial navigation. (**A**) A schematic illustration shows a freely moving mouse with the head-mounted navigation system for egocentric stimulus-based learning in the double Y-maze. (**B**) The percentage of correctness (upper) and the total number of visited goal points until finding the correct goal (lower) for each block within the training period (10 blocks; 36 trials in a block). Data from the trained group of mice (n = 7/10) was used for analysis. The bar graphs show that behavioral performance significantly declines when no egocentric stimulus was provided (blue bar, with egocentric stimuli; gray bar, without egocentric stimuli; n = 7, two-tailed paired t-test, p = 0.004 in upper, p = 0.007 in lower). (**C**) A comparison of the percentage of correctness and the number of passing goal points between trained (n = 7/10, unfilled bar) and untrained mice (n = 3/10, filled bar). There was no significant difference between the two groups during the first training block (two-tailed t-test, n = 7 vs. 3, p = 0.718 in left, p = 0.634 in right). In contrast, significant differences were detected for the tenth block (two-tailed t-test, n = 7 vs. 3, p = 0.030 in left, p = 0.020 in right). *p < 0.05; n.s., no significant differences; error bars represent the mean ± SEM.

To examine whether animals can use egocentrically-stable cues during spatial navigation, we tested whether visual cues facilitate path finding in a double Y-maze for mice (Fig. 1A). The egocentric stimuli were provided through light illumination from two head-mounted LEDs. The LED corresponding to the correct direction the mice had to turn towards was illuminated just before every fork along the correct path and was turned off after the mice entered the fork. Since the LEDs were head-mounted, the illumination of the LEDs served as an egocentrically stable cue, indicating the correct direction to a specific goal point. To rule out the possibility that the mice used other strategies independent of the provided egocentric stimuli and that there was any bias towards any of the goal points, we designed the experiment as follows: 1) We used the computer-controlled pellet dispenser to reward the mice without human intervention. 2) The goal point was randomly assigned in every trial. 3) The whole maze was randomly rotated after every trial. Using this experimental paradigm, we trained the mice to use the egocentrically stable LED cues to find the goal area.

### Mice successfully learn egocentric stimulus-based spatial navigation

For the quantitative analysis of egocentric stimulus-based learning, we measured the ratio of correct decisions made at the forks over all trials. After 30 days of training, we found that 70% of the mice (trained group, n = 7/10 mice) exhibited a significant increase in correctness (1st block, 46.9 ± 1.5%; 10th block, 70.0 ± 1.8%), whereas the other mice (untrained group, n = 3/10 mice) did not show significant progress in learning (1st block, 45.8 ± 1.4%; 10th block, 57.4 ± 2.8%; Supplementary Fig. 1). We also found that the performances of the trained mice significantly decreased when egocentric stimuli were eliminated (n = 7/10; with egocentric stimuli, 72.0 ± 2.7%; without egocentric stimuli, 42.9 ± 4.5%; Fig. 1B), which suggests that the increase in correctness is dependent on the use of egocentric stimuli. Furthermore, we tested whether successful learning with egocentric stimuli led to a more efficient navigational strategy. Measuring the number of visited goal points in each trial (all incorrect goal points that were visited in addition to the correct goal point), we found that the trained mice passed a significantly fewer number of points (1.7 ± 0.1 points) compared to untrained mice (2.2 ± 0.0 points, Fig. 1C). As expected, for the trained mice group, the number of visited goal points significantly decreased over the course of the training session (1st block, 4.7 ± 0.2 points; 10th block, 1.7 ± 0.1 points; Fig. 1B) and significantly increased when egocentric stimuli were eliminated (with egocentric stimuli, 1.8 ± 0.1 points; without egocentric stimuli, 4.0 ± 0.5 points; Fig. 1B). Collectively, we found that mice could learn to use egocentric stimuli for spatial navigation within 30 days of training, which suggests that our experimental paradigm is a promising model to study egocentric stimulus-based learning in animals.

### Sigmoidal learning progress for egocentric stimulus-based spatial navigation task

**Figure 2:**
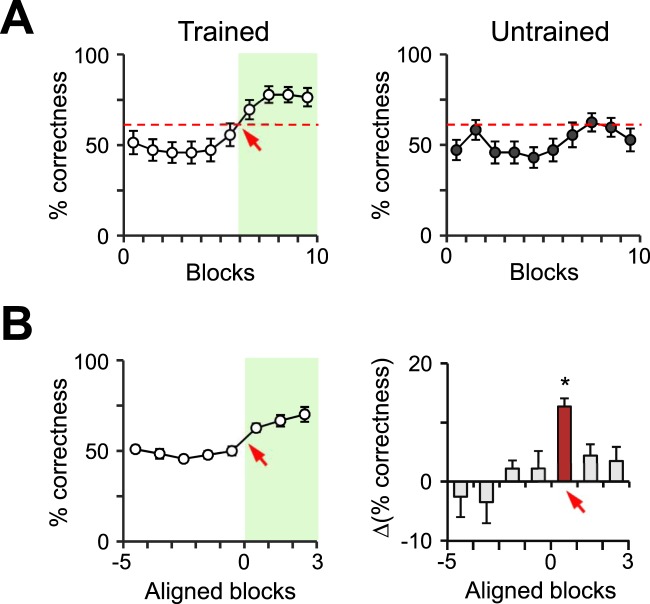
Sigmoidal learning curves in egocentric stimulus-based learning. (**A**) The learning curves of representative mice for the trained (unfilled) and untrained (filled) groups. The red dashed line indicates the lowest possible percentage that would signify a statistically significant amount of correct choices. The red arrow indicates the Eureka point, and the period after the Eureka point is shaded in green. (**B**) Left: The line graph shows the average learning curve of the trained mice (n = 7/10) by aligning the data with the Eureka point of each mouse. The red arrow and green shading match the description from panel A. Right: The bar graph shows differences of correctness between the two consecutive blocks. The value of the Eureka point elicited a significant difference when compared with two adjacent points (red bar vs. gray bars; one-way ANOVA, F = 6.396, p = 0.008, there is a significant difference in pairwise comparison procedures as determined by Tukey’s method). *p < 0.05; n.s., no significant differences; error bars represent the mean ± SEM.

A learning curve represents the pattern of learning based on experiences over time. There are three typical curves that represent successful learning: exponential, logarithmic, and sigmoidal. The exponential curve indicates gradual increment of learning without limit, whereas the logarithmic curve represents rapid learning during an initial period followed by a gradual decline of the learning speed, with performance reaching a saturation point. The sigmoidal curve begins as an exponential pattern but later transforms into a logarithmic pattern. In other words, learning starts slowly, improves rapidly, and then reaches saturation. Interestingly, our model of orientation-based learning exhibited a sigmoidal curve with a long latent period before the “Eureka” point. Once this point was reached, a drastic increase in the ratio of correct choices occurred, which was then followed by a performance plateau (Fig. 2A and Supplementary Fig. 1; Experimental procedures).

To confirm whether this learning curve exhibited any patterns of the typical curves mentioned above, we calculated the difference between the average percentages of correctness for every pair of consecutive blocks. Comparison of these values revealed that only one period showed a significantly higher level of learning. (Fig. 2B). This result suggests that egocentric stimulus-based learning follows a sigmoidal growth pattern characterized by a sufficient preparatory period of slow learning and a fast learning stage that eventually levels off. This learning process may be functionally significant for collecting, learning, and retaining information and consistent with the concept of latent learning.

### External route mark-based learning requires a shorter preparatory period than egocentric stimulus-based learning

**Figure 3:**
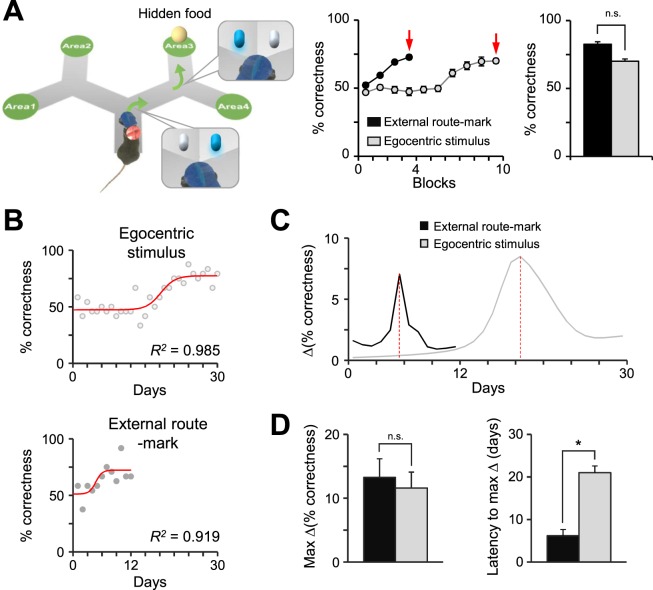
External route mark-based learning is more rapidly attained compared to egocentric stimulus-based learning. (**A**) A schematic illustration of the external route mark-based learning paradigm. The line graph shows the ratio of correctness during the training periods for egocentric stimulus-based learning (gray) and external route mark-based learning (black). Data from the trained group (n = 5/10 for external route mark-based learning, 7/10 for egocentric stimulus-based learning) were used for analysis. The red arrows indicate the last training block in each paradigm, and no significant difference in the percent of correctness was shown between external route mark- and egocentric stimulus-based learning (bar graph; n = 5 vs. 7, two-tailed t-test, p = 0.529). (**B**) The representative data from the egocentric stimulus- and external route mark-based learning paradigms fitted with sigmoidal curves. The red lines show the fitted curve with logistic functions, and the scatter plots show the average percentage of correctness on each day. The R-squared value indicates the coefficient of determination. (**C**) The line plots illustrate the amount of change in the percentage of correctness during the training period (black, external route-mark; gray, egocentric stimulus). The red dashed line indicates the time at which the maximum amount of change was achieved. (**D**) Left: The maximum amount of change between the external route-mark and egocentric stimulus paradigms showed no significant difference (n = 5 vs. 7, two-tailed t-test, p = 0.423). Right: The latency to reach the maximum amount of change was significantly different between the external route-mark and egocentric stimulus paradigms (n = 5 vs. 7, two-tailed t-test, p = 0.011). *p < 0.05; n.s., no significant differences; Error bars represent the mean ± SEM.

Despite the fact that the head-mounted cues were stable in egocentric space, we initially questioned whether the mice perceived these cues to be different from allocentric ones. The egocentrically stable discriminative stimuli we provided during the experiment do exhibit allocentric spatial characteristics when the mouse is approaching a fork, as the LED that is illuminated coincides with the direction of the corresponding pathway in the fork. To test whether mice perceive these cues differently, we conducted an external route mark-based learning experiment in the same maze with maze-mounted LED lights on the walls of the fork instead of the head-mounted device (Fig. 3A). The mice reached the plateau percentage of correctness within 12 days of training (Fig. 3A; Supplementary Figs 2 and 3), which was comparatively shorter than the 30 days it took for the mice that navigated with the egocentric stimulus-based protocol. However, there were no significant differences both in the maximum asymptotic performance in the last training block (Fig. 3A) and in the ratio of trained to untrained mice (50% in external route-marks vs. 70% in egocentric stimuli, Fisher’s exact test, p = 0.650).

In addition, we compared the learning curve of the external route mark-based paradigm with that of the egocentric stimuli-based paradigm through regression analysis. Interestingly, the learning curves for both paradigms showed a sigmoidal growth pattern despite the different latent learning periods (Fig. 3B). This result indicates that navigational strategies that use either egocentric stimuli or external route-marks manifest a similar learning process. However, external route mark-based learning can be more rapidly facilitated compared to egocentric stimulus-based learning. Moreover, to examine the differences between the two curves, we compared the factors associated with the sigmoidal shape of the curves. Remarkably, we found that there was a significant difference in the latency to reach the respective maximum learning speed for the egocentric stimulus- (17.4 ± 2.7 days) and the external route mark- (6.3 ± 1.1 days) based learning, but no significant difference between the maximum learning speed themselves in egocentric stimulus- (11.6 ± 2.5%) and external route mark- (13.3 ± 2.9%) based learning (Fig. 3C and D). Collectively, these results suggest that the crucial difference between egocentric stimulus- and external route mark-based learning is the duration of the preparatory learning period that precedes the initiation of fast learning.

### Biased selection strategies independent of discriminative stimulus-based learnings

**Figure 4:**
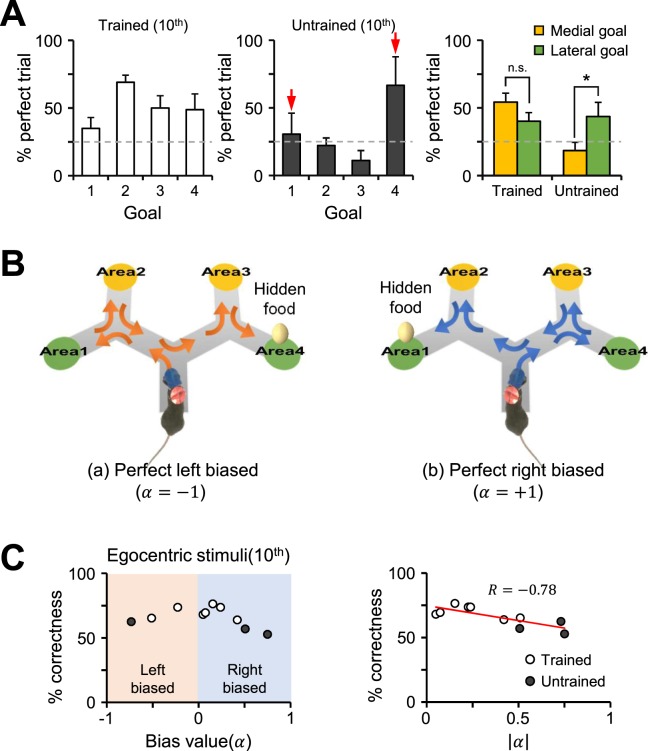
A biased selection strategy negatively correlates with egocentric stimulus-based learning. (**A**) The proportion of perfect trials among the trials with the same correct goal point for both the trained (unfilled, n = 7/10) and untrained (filled, n = 3/10) mice in the egocentric stimulus-based learning group. The gray dashed line indicates the probability of randomly choosing the correct goal point (one out of four). The red arrows indicate the two goals that are lateral. The proportions of perfect trials for the trials with a medial goal point as the assigned destination (areas 2 and 3, yellow) and those with a lateral goal point as the assigned destination (areas 1 and 4, green) were compared. The trained mice showed no significant differences on the percentage of correctness between the medial and lateral trials (n = 7, two-tailed paired t-test, p = 0.189), whereas the untrained mice show a significantly lower value for the medial trials compared to the lateral ones (n = 3, two-tailed paired t-test, p = 0.047). (**B**) The illustrations show an example of a mouse using a biased direction strategy (orange arrows in the left panel, left bias; blue arrows in the right panel, right bias). The alpha value indicates the amount of bias. (**C**) Left: The scatterplot shows the percentage of correctness versus the bias value for both the trained (unfilled) and untrained (filled) groups (orange shadow, left-biased selection; blue shadow, right-biased selection). Right: The percentage of correctness versus the absolute value of the bias value. The red line is the best fit line resulting from linear regression, and the R-value indicates Pearson’s correlation coefficient. *p < 0.05; n.s., no significant differences; Error bars represent the mean ± SEM.

Although we controlled the experimental conditions in an attempt to prevent mice as much as possible from learning some unintended navigation strategy, we thought the possibility could not be completely ruled out. The results confirmed our suspicions as we found that the untrained group of mice showed a higher success rate when the goal point was assigned in the lateral part of the maze both in egocentric stimulus-based learning (n = 3; medial, 18.6 ± 6.4%, lateral, 43.7 ± 11.1%; Fig. 4A) and in external route mark-based learning (n = 5, medial, 20.7 ± 6.4%, lateral, 45.7 ± 4.4%; Supplementary Fig. 4). Because the optimal route towards lateral goal points involves two consecutive turns in the same direction, we predicted this result to be the consequence of a biased selection strategy that chooses the same direction at every fork. To test this possibility, we measured each mouse’s level of bias with a bias value (α), which was calculated by mapping the proportion of turns in the same direction to an index between -1 (choose the left path at all forks; perfect left biased) and 1 (choose the right path at all forks; perfect right biased, Fig. 4B). We then analyzed whether the level of bias correlates with learning progress in both the egocentric stimulus- and the external route mark-based protocols. Remarkably, we found that there is a significantly negative correlation between the absolute value of the bias index and the ratio of correctness (Fig. 4C and Supplementary Fig. 4). Although the trained group of mice exhibited low levels of bias, their respective results showed the same trend. As a result, we found that biased selection is a possible strategy for mice during spatial navigation, competing with the egocentric stimulus- and the external route mark-based strategies.

## Discussion

Our study confirmed that mice have the ability to perform spatial navigation using egocentrically-stable discriminative stimuli as routing information. Although egocentrically given visual instructions for navigation are easily used in our daily life, application of these navigational strategies in animals has been a challenge due to the difficulty of providing consistent and reproducible visual signals to freely moving animals. By implementing a head-mounted device for mice, our finding provides the first evidence that rodents can learn to use route information from the egocentrically-stable visual orientation cues. Although previous studies reported that several social animals can understand pointing cues for a choice task^7–9^, it is different from our study where the cues are egocentrically fixed with respect to the mouse’s head. Moreover, our experimental protocol can be easily adopted by other studies with various species because it utilizes a simple electrical system that requires no involvement from the investigator. This method also addresses the conflict surrounding domestication efforts in previous instruction learning experiments^9,10^. Due to the broad and flexible range of applications, it can be used for high-throughput studies such as phenotyping mutations or comparing interspecies differences.

To examine the differences between the performances of external route mark- and egocentric stimulus-based paradigms and to focus on the progress of learning across repeated trials, mice were given a delayed reward for completing a double Y-maze task. The results obtained by providing a delayed reward showed that the trained group of mice showed a drastic increase in performance for both external route mark- and egocentric stimulus-based learning, which is in line with the classical idea of single-trial learning^11,12^. The learning curve for the trained mice exhibited a sigmoidal shape, which is often found in many different learning paradigms, and several mathematical approaches such as dynamic interval determination and cumulative calculation can be utilized for a detailed analysis of such learning curves^13^. Furthermore, the results in our study show that external route mark-based learning shows better performance of learning than the egocentric stimulus-based learning used in our paper, considering the Eureka points of the two learning curves. On the other hand, having no significant difference in the maximum learning speed and the maximum asymptotic performance for both paradigms may indicate that there exist similarities between the two. This may be because of the allocentric spatial characteristics that our egocentric stimulus-based paradigm may have, especially in the case where the mouse approaches a fork facing forward (Figs 1A and 3A). Further studies on these hypotheses may be conducted.

In our experiment, the untrained mice that were unable to adapt to our visual stimuli did not use the information from the cues but eventually found the goal using a simple strategy, i.e., turning to the same direction at every branch point (Fig. 4). This behavior can be explained by thigmotaxis, which is the natural tendency of rodents to remain close to vertical surfaces^14^. In short, the mice just followed the walls until they arrived at the goal area. Although the biased choice strategy failed to utilize the shortest path to the goal, it is still an effective strategy for mazes with fixed spatial patterns and with a different goal location on each trial. Despite a low level of correctness, the group of untrained mice showed shorter trial durations and total distances to a goal after the training session for both egocentric stimulus- and external route mark-based learning (Supplementary Figs S5 and S6). However, when exposed to an unknown and more complex maze or environment, this strategy may be less effective and the mice may start to rely on cue-based navigation. This conditional strategy hypothesis may link to the idea that animals use different navigational strategies depending on the complexity of the environment, which is an interesting idea for further studies using different types of mazes.

Although the hippocampus is a critical brain region for spatial learning^15–17^, understanding route information from the discriminative stimulus may depend on other neural pathways, such as frontal cortical areas, that account for decision-making with sensorimotor integration^18–20^. This hypothesis can be tested in future studies using neural circuit modulation techniques, such as optogenetics^21,22^. These techniques can help elucidate how visual cues are interpreted as routing information in the brain. In summary, we investigated the behavioral paradigms of mice while applying two different navigational strategies: one using external route-marks and the other using egocentrically stable discriminative cues. By comparing the learning curves for the two aforementioned visual cues, our finding supports that mapping an egocentric cue to an egocentric action showed slower learning progress than mapping the egocentric action to an allocentric cue, although both paradigms achieved similar performance in the long run. Further studies with optogenetic methods and genetic mouse models can be applied to provide insight on what the differences in neural mechanisms are while performing navigation tasks with the two aforementioned strategies.

## Methods

### Animal care and surgery

All experimental protocols and animal care were performed in accordance with the institutional guidelines and approved by the Animal Care and Use Committee of Korea Advanced Institute of Science and Technology (Protocol No. KA2014-05). All mice were maintained on a 12:12 hour light:dark cycle (with the light cycle beginning at 6:00 AM) and kept in a room whose temperature was maintained at 23 °C. Food and water were supplied ad libitum. All laboratory animals used in this experiment were male C57B/L6 mice with ages ranging from 2 to 6 months old. During the experimental period, which restricted the mice’s access to food, the body weights of the mice did not fall below 80% of their initial weights, which were measured at the beginning of the experimental period. For fixing the LED hat onto the mice’s heads, a head-plate was attached to the top of the skull of each mouse via stereotaxic surgery. During the surgery, mice were anesthetized with avertin (20 mg/ml of tribromoethanol, 20 µl/g i.p.) and placed in a stereotaxic apparatus (David Kopf Instruments, USA). Super-Bond (Sun Medical, Japan) was used to fix the head-plate onto the skull.

### Experimental apparatus for egocentric stimulus-based learning

The LED hat is a core element of our egocentric stimulus-based navigation system. It consists of two LEDs (3 mm flat top cylindrical blue LEDs; powered by 3.2 V, 20 mA), a Bluetooth communication module (RFduino-DIP, SparkFun Electronics, USA; 3 V, 18 mA), a battery (H501430-PCM, Power Source Energy, Taiwan; 3.6 V, 170 mAh), and colored markers. The LED hat provides information about the correct choice at a fork during the double Y-maze experiments by lighting up the LED in the same direction as the correct path. The Bluetooth communication module was used to wirelessly control the illumination of the two LEDs. The colored markers served as indicators for recognizing the positions and orientations of the mice during the experiments. As a whole, the hat is 2.0 cm × 4.0 cm in size and weighs 5.0 g. Due to the small size and low weight, the hat did not affect the movements of the mice. The hat was fixed onto the head-plates that were surgically attached to the mice’s skulls.

### Maze structure

The double Y-maze was created with black acrylic plate to minimize any other visual cues, and the maze was cleaned with alcohol in between each experiment to prevent the mouse from recognizing the position of the maze using olfactory information. Each corridor of the double Y-maze was created to be 0.1 m in width and 0.2 m in length. To prevent the mouse from being distracted by the external environment while within the maze, the walls were created to be 0.15 m in height. Each goal point also has an automatically controlled custom-made pellet dispenser, which was used to reward the mice whenever they successfully arrived at the correct goal point. In addition, wall-mounted LEDs were installed on the walls before each fork and were used to provide external route-mark cues.

The maze was created with an automated system such that human interference was not needed (i.e. taking out a mouse from the maze and placing it somewhere else for the next trial). To be more specific, in the first trial, an outer fork connecting two consecutive goal points and those two goal points were selected to be the starting area. Then, out of the remaining goal points, a destination was randomly selected. In the following trial, the goal point the mouse chose in the previous trial, the goal point adjacent to the chosen goal point, and the fork connecting those two goal points were considered to be the new starting area. A new destination goal point was then randomly chosen out of the remaining goal points. This pattern was iterated throughout all consecutive trials of one mouse subject.

The maze was placed in a dark room, and four light bulbs (OSRAM Dulux EL, 20 W/860) were fixed above it to maintain consistent brightness throughout the maze.

### Habituation and behavioral training

After surgery, all mice went through a process of habituation that lasted three days to adapt to the LED hat and head-plate, the learning space, the maze, and the custom-made pellet dispenser feeding system. During the habituation process, all mice were able to move freely within the maze with the hat attached, and no cues were provided. To teach the mice about the existence of rewards in the maze, a food pellet (Bio-serv, USA; 20 mg) was automatically provided through a pellet dispenser whenever the mice arrived at a randomly assigned goal point. After the habituation process, a training period for egocentric stimulus-based learning and external route mark-based learning was held for 30 days and 12 days, respectively. All mice for both types of learning were subjected to 12 training trials a day. After the training period, testing was conducted for a period of 2 days. Out of the 12 test trials each per testing day for both egocentric stimulus- and external route mark-based learning, cues were provided for six trials, and no cues were given for the remaining trials.

### Computer program for real-time tracking, data collecting, and centralized control of the maze during testing

All experimental sessions were recorded with a digital camcorder (LifeCam HD-6000, Microsoft, Microsoft, USA) placed above the maze. The camcorder transferred frames from the video footage in real-time to a computer system that autonomously tracked the mice and controlled the LEDs. Within the computer system, each fork (central area in a Y-maze) was detected as a triangular zone in the transferred frames. The computer system autonomously determined whether the mice were approaching a fork by detecting the distance between the colored marker on the mice’s head-mounted devices and the triangular zone of the incoming fork. For egocentric stimulus-based learning, egocentric stimuli were provided through the LEDs to indicate the correct direction only when a mouse approached a fork. The LED was turned on when the distance between the mouse (the colored marker on its hat) and fork (the closest edge of the triangular zone to the mouse) was under 5 cm and turned off immediately after the mouse entered the fork. If the mice failed to select the correct path during a trial, no egocentric stimulus was provided until mice reached any of the incorrect goal points. After the mouse turned back from the incorrect goal point and started to move to another location, egocentric stimuli were provided towards the correct goal point assigned at the beginning of the trial. The procedure was repeated until the mouse found the correct goal point, at which point the trial was completed. The computer system was programmed with MATLAB (Mathworks, USA), C, and Python. A snapshot of a mouse with the head-mounted LED device is provided in Supplementary Fig. 7.

### Data analysis

10 mice each were prepared for the egocentric stimulus-based experiment and the external route mark-based experiment. However, the performances of three mice in the egocentric stimulus group and five mice in the external route-mark group did not improve in a statistically significant manner at the 95% confidence level, displaying insufficient learning. Therefore, we analyzed the results of learning for the trained groups and untrained mice separately. The criterion for the classification of the mice as either trained or untrained is whether the mice showed a statistically significant increase in performance during the last 3 training days compared to the first 3 training days. The Eureka point was determined to be the time point that showed the most drastic and statistically significant increment during the training session. For regression analysis of the learning curves, we used logistic functions provided in MATLAB.

## Supplementary information

Supplementary Figures
