# Supplementary material for: Egocentrically-stable discriminative stimulus-based spatial navigation in mice: implementation and comparison with allocentric cues
Source: Sci Rep. 2019 Apr 23;9:6451. doi: 10.1038/s41598-019-42852-0 (PMC6478847; doi:10.1038/s41598-019-42852-0)
Supplement: Supplementary file 1 — Supplementary Figures [file 41598_2019_42852_MOESM1_ESM.docx]

Supplementary information

**Egocentrically-stable discriminative stimulus-based spatial navigation in mice: implementation and comparison with allocentric cues**

Jinsung Chun, Youngsoo Kim, Jin Woo Choi, Daesoo Kim and Sungho Jo

- Supplementary figure 1
- Supplementary figure 2
- Supplementary figure 3
- Supplementary figure 4
- Supplementary figure 5
- Supplementary figure 6
- Supplementary figure 7
- Supplementary figure 8

**
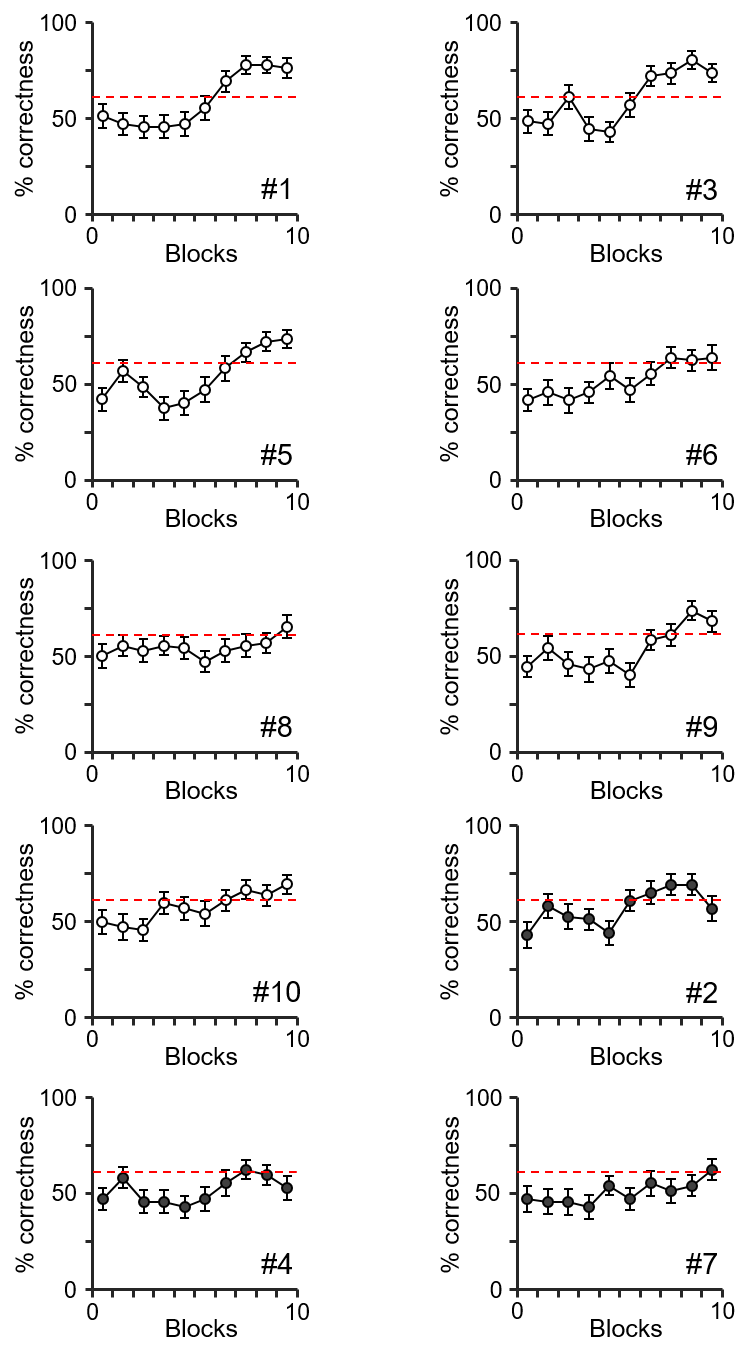
**

**Figure S1. The progress of egocentric stimulus-based learning in an individual mouse.**

The percentage of correctness during the training period of egocentric stimulus-based learning for each mouse (unfilled points, trained group; filled points, untrained group). The red dashed line indicates the lowest possible percentage that would signify a statistically significant amount of correct choices.


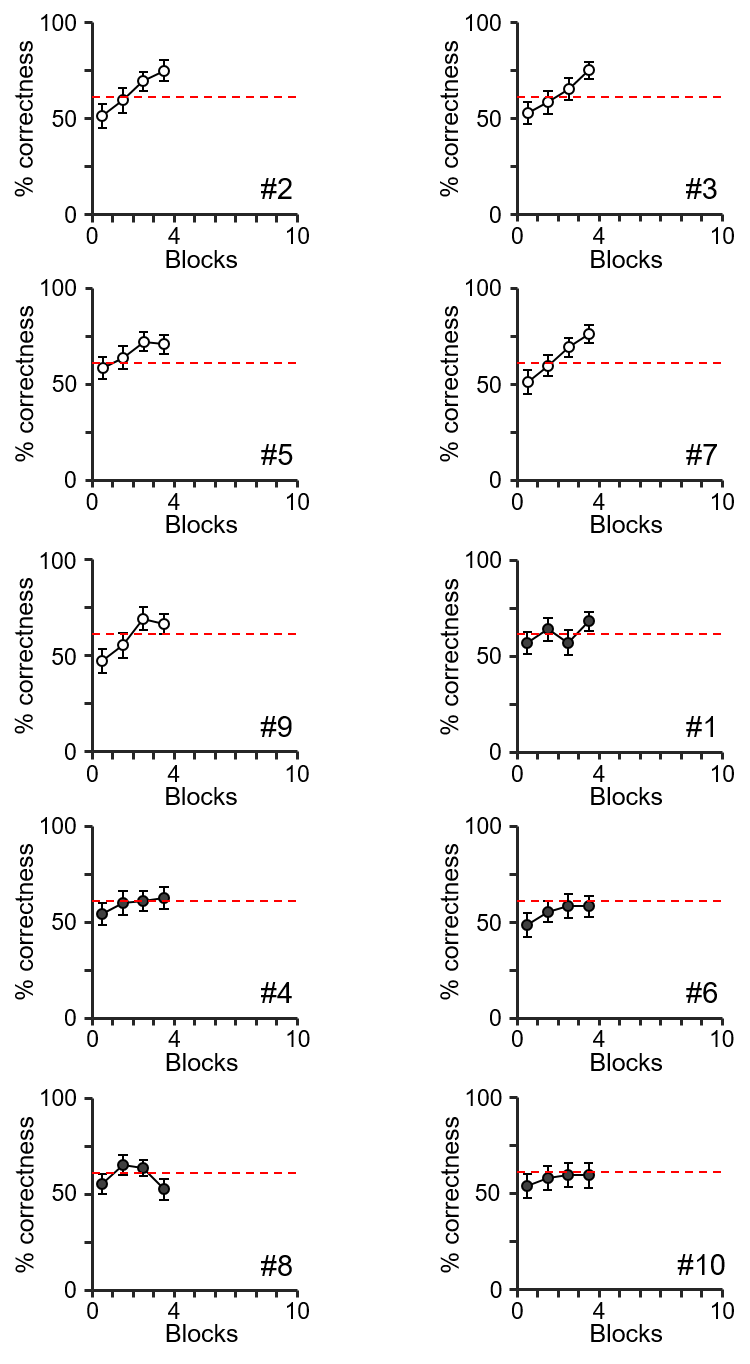


**Figure S2. The progress of external route mark-based learning for each mouse.**

The percentage of correctness during the training period of external route mark-based learning for each mouse (unfilled points, trained group; filled points, untrained group). The red dashed line indicates the lowest possible percentage that would signify a statistically significant amount of correct choices.

**
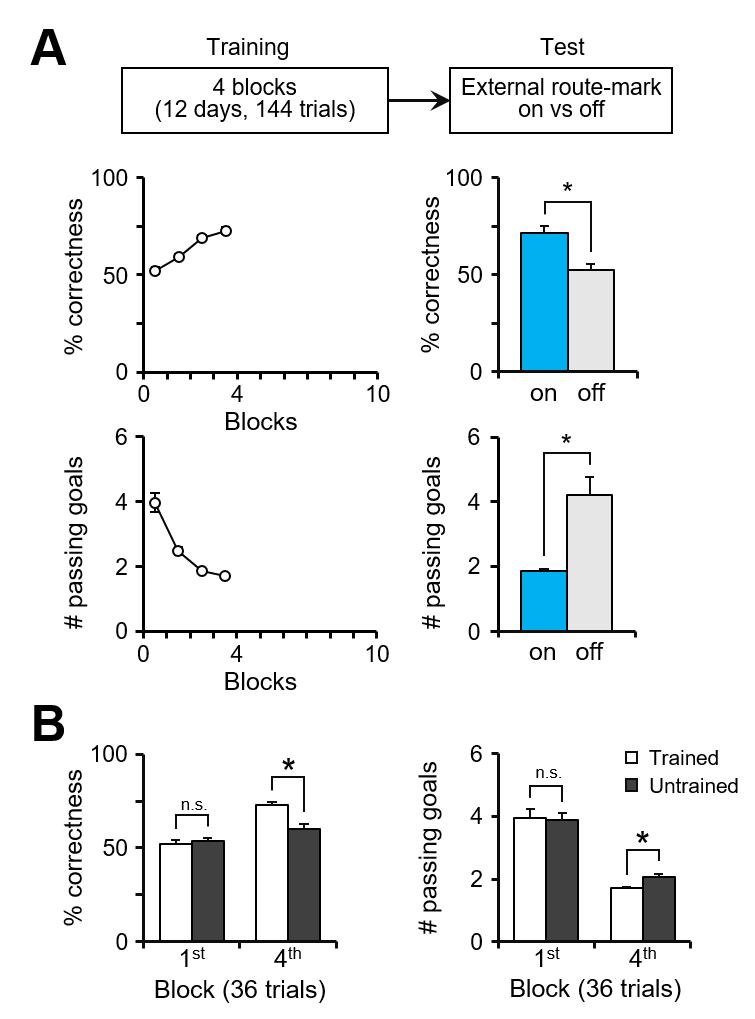
**

**Figure S3. Mice successfully facilitate external route mark-based based learning for spatial navigation.**

(A) The percentage of correctness (upper) and the total number of visited goal points until finding the correct goal (lower) for each block within the training period (4 blocks; 36 trials in a block). Data from the trained group of mice (n = 5/10) was used for analysis. The bar graphs show that behavioral performance significantly declines when lacking external route-marks (blue bar, with external route-mark; gray bar, without external route-mark; n = 5, two-tailed paired t-test, p = 0.016 in upper, p = 0.044 in lower). (B) A comparison of the percentage of correctness and the number of passing goal points between trained (n = 5/10, unfilled bar) and untrained mice (n = 5/10, filled bar). There was no significant difference between the two groups in the first block (two-tailed t-test, n = 5 vs. 5, p = 0.607 in left, p = 0.841 in right), in contrast to the significant difference in the fourth block (two-tailed t-test, n = 5 vs. 5, p = 0.015 in left, p = 0.010 in right).

*p < 0.05; n.s., no significant differences; Error bars are represented as mean ± SEM.


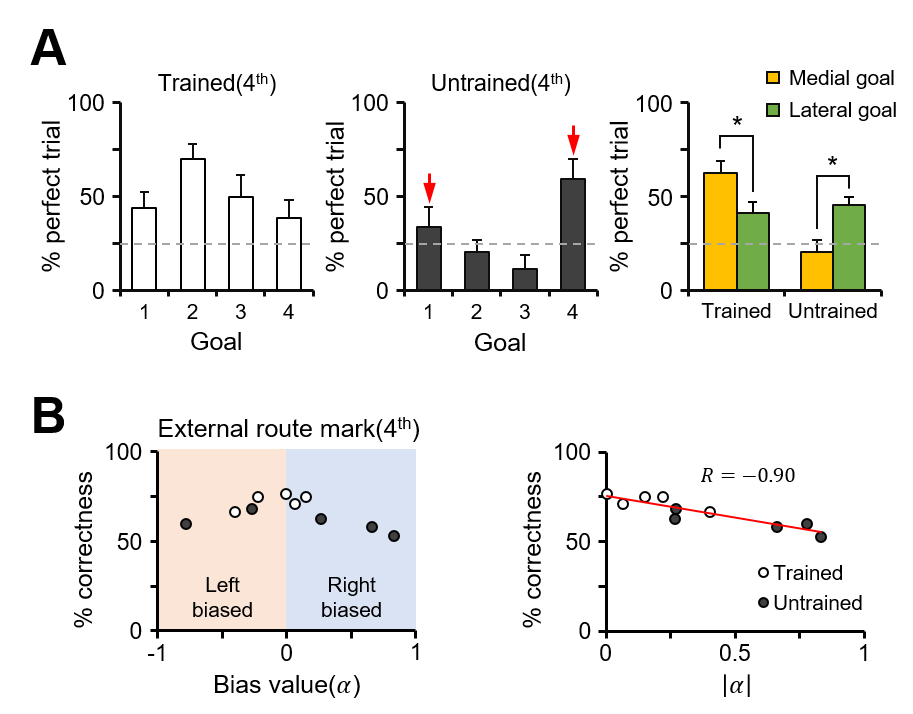


**Figure S4. Biased selection strategy is negatively correlated with external route mark-based learning.**

(A) The proportion of perfect trials among the trials with the same correct goal point for both the trained (unfilled, n = 5/10) and untrained (filled, n = 5/10) mice in the external route mark-based learning group. The gray dashed line indicates the probability of randomly choosing the correct goal point (1 out of 4 or 25%). The red arrows indicate the two goals that are lateral. The proportions of perfect trials for the trials with a medial goal point as the assigned destination (area 2 and 3, yellow) and those with a lateral goal point as the assigned destination (area 1 and 4, green) were compared. The trained mice show a significantly higher percentage of correctness for the medial trials compared to the lateral ones (n = 5, two-tailed paired t-test, p = 0.035), whereas the untrained mice show a significantly lower value for the medial trials compared to the lateral ones (n = 5, two-tailed paired t-test, p = 0.007). (B) Left: The scatterplot shows the percentage of correctness versus the bias value for both the trained (unfilled) and untrained (filled) groups (orange shadow, the case with left biased selection; blue shadow, the case with right biased selection). Right: The percentage of correctness versus the absolute value of the bias value. The red line is the best fit line resulting from linear regression, and the R-value indicates Pearson’s correlation coefficient.

*p < 0.05; n.s., no significant differences; Error bars are represented as mean ± SEM.


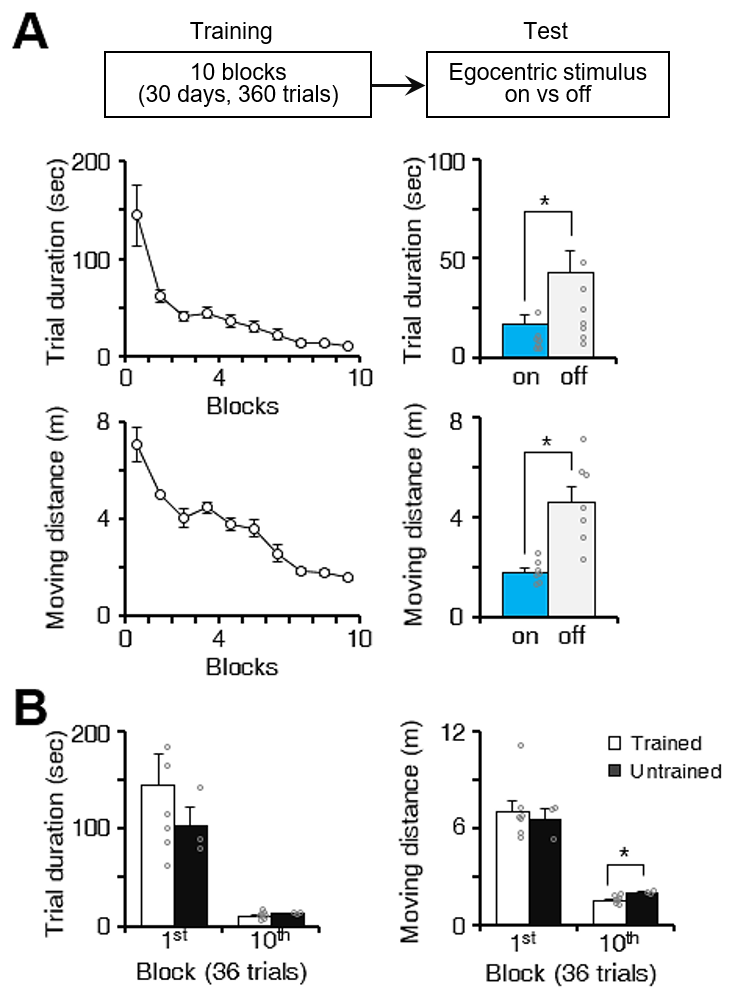


**Figure S5, related to Figure 1. Latency to reach the goal and total path length of mice during egocentric stimulus-based learning.**

(A) The bar graphs show that behavioral performance declined when egocentric stimuli were not provided (blue bar, with egocentric stimuli; gray bar, without egocentric stimuli; n = 7, two-tailed paired t-test, p = 0.013 in upper, p = 0.004 in lower). (B) The trial duration and the moving distance in a trial were compared between the trained (n = 7/10, unfilled bar) and untrained mice (n = 3/10, filled bar). There was no significant difference between the two groups in the first training block (two-tailed t-test, n = 7 vs. 3, p = 0.289 in left, p = 0.636 in right), whereas in the tenth block, there was a significant difference in the moving distances between the untrained and trained groups, but not in trial duration (two-tailed t-test, n = 7 vs. 3, p = 0.197 in left, p = 0.002 in right). *p < 0.05; n.s., no significant differences; Error bars represent the mean ± SEM.


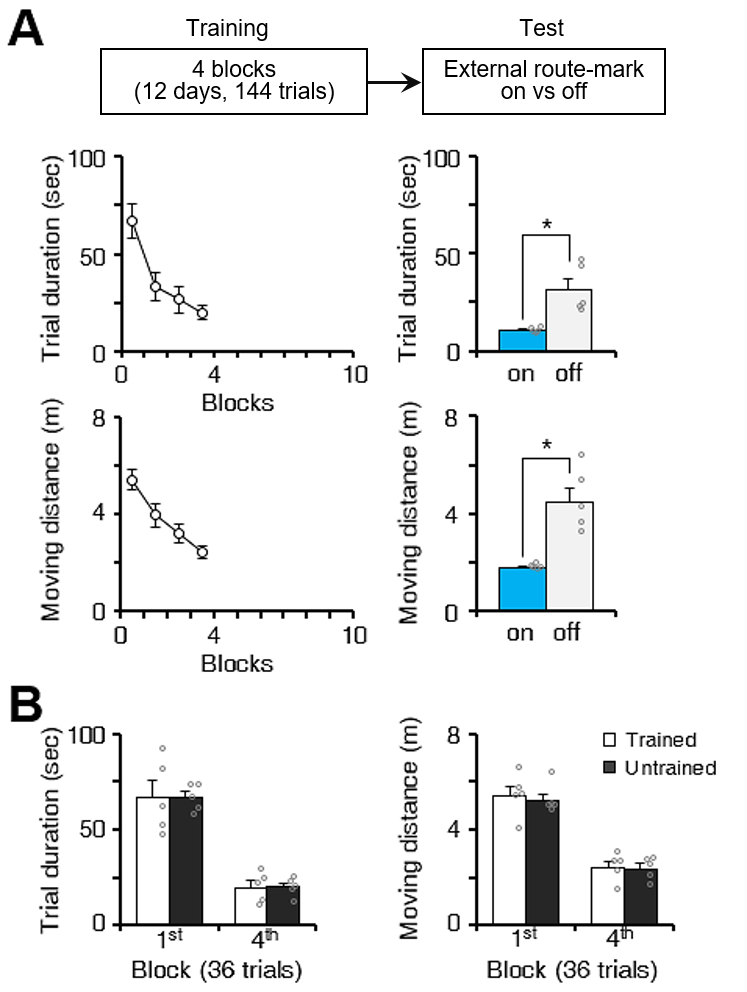


**Figure S6, related to Figure 3. Latency to reach the goal and total path length of mice during external route mark-based learning.**

(A) The bar graphs show that behavioral performance declined without landmarks (blue bar, with external route-mark; gray bar, without external route-mark; n = 5, two-tailed paired t-test, p = 0.022 in upper, p = 0.010 in lower). (B) The trial duration and the moving distance in a trial were compared between the trained (n = 5/10, unfilled bar) and untrained mice (n = 5/10, filled bar). There were no significant differences between the two groups both in the first training block (two-tailed t-test, n = 5 vs. 5, p = 0.956 in left, p = 0.731 in right) and fourth block (two-tailed t-test, n = 5 vs. 5, p = 0.972 in left, p = 0.863 in right). *p < 0.05; n.s., no significant differences; Error bars represent the mean ± SEM.


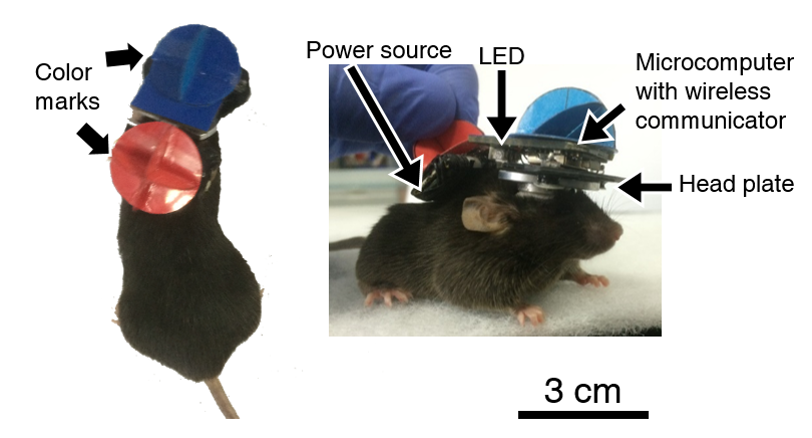


**Figure S7. A snapshot of a mouse with the head-mounted device.**

A snapshot of the head-mounted LED device on a mouse.


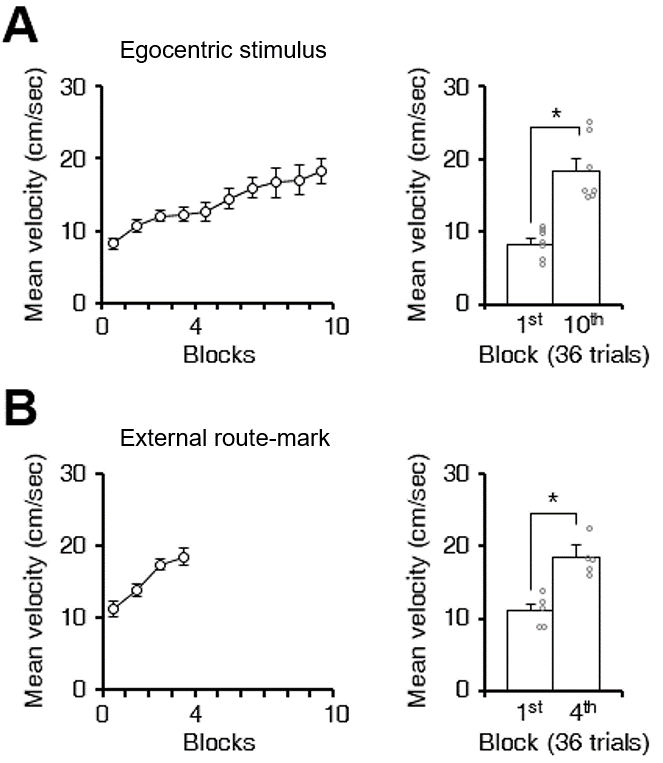


**Figure S8, related to Figure 1 and 3. Mean velocity in a trial during external route mark-based learning of mice.** (A) The mean velocity for each block within the training period for egocentric stimulus-based learning (10 blocks; 36 trials in each block). Data from the trained group (n = 7/10) was used for analysis. The bar graph shows that the mean velocity significantly increased from the first block to the tenth block. (1st vs. 10th; n = 7, two-tailed paired t-test, p < 0.001). (B) The mean velocity for each block within the training period for external route mark-based learning (4 blocks; 36 trials in each block). Data from the trained group (n = 5/10) was used for analysis. The bar graph shows that the mean velocity significantly increased from the first block to the fourth block. (1st vs. 4th; n = 5, two-tailed paired t-test, p = 0.004). *p < 0.05; n.s., no significant differences; Error bars represent the mean ± SEM. Gray dots represent individual data point.
